# Coelomic Fluid of* Lumbricus rubellus* Synergistically Enhances Cytotoxic Effect of 5-Fluorouracil through Modulation of Focal Adhesion Kinase and p21 in HT-29 Cancer Cell Line

**DOI:** 10.1155/2019/5632859

**Published:** 2019-04-11

**Authors:** Agustina Tri Endharti, Yeni Purnamasari, Renata Primasari, Sri Poeranto, Sofy Permana

**Affiliations:** ^1^Department of Parasitology, Faculty of Medicine, Brawijaya University, Malang, Indonesia; ^2^Biomedical Central Laboratory Faculty of Medicine, Brawijaya University, Malang, Indonesia; ^3^Doctoral Program in Biomedical Sciences, Faculty of Medicine, Brawijaya University, Indonesia; ^4^Magister in Biomedical Science Program, Medical Faculty, Brawijaya University, Malang, Indonesia; ^5^Department of Biology, Faculty of Mathematics and Natural Sciences, Brawijaya University, Malang, Indonesia

## Abstract

Coelomic fluid of* Lumbricus rubellus* (CFL) has attracted interest due to its pharmacological properties, including antitumor effect. Furthermore, it is necessary to evaluate the response to treatment with new cancer therapeutic agents. This study aims to investigate whether the combination of CFL and 5-fluorouracil could reduce FAK protein level and iCa^2+^ and enhance p21 level. Furthermore, it is necessary to evaluate the response to treatment with new cancer therapeutic agents. After 24 hours of treatment, it was necessary to assess the percentage of apoptosis, FAK, and p21 protein expression by flow cytometry. iCa^2+^ concentration was measured using immunofluorescence. The combination therapy of CFL with 5-fluorouracil potently suppressed six treatment groups were included in this study. HT-29 cell lines were cultured and divided into six groups: group 1 was treated with vehicle (negative control), groups 2-5 were treated with 5-fluorouracil, groups 3-5 were treated with either CFL 5, 10, or 20 *µ*g/ml immediately after 5-fluorouracil, and group 6 was treated with CFL 20 *µ*g/ml, the progression of colorectal cancer. Combination of CFL and 5-fluorouracil significantly decreased FAK expression (p<0.05), iCa^2+^ (p<0.05), and increased p21 expression (p<0.05) in HT-29 cells. Our results suggest that CFL has an anticancer potential in colorectal cancer when combined with 5-fluorouracil.

## 1. Introduction

Cancer is one of the leading causes of death worldwide. Colorectal cancer is one of the most common malignancies with a high prevalence and low 5-year survival rate [1]. The incidence of colorectal cancer as well as the mortality rate is very high. Chemotherapy allows for the treatment of patients with advanced-stage tumors or those who have relapsed. 5-Fluorouracil (5-FU) is a cytostatic drug used for the treatment of various types of cancers especially colon cancer [2]. An antimetabolite chemotherapy agent such as 5-FU has a primary function to convert thymidylate synthetase (TS) into deoxythymidine monophosphate (dTMP) in cells that are actively proliferating [3]. However, due to the high intrinsic resistance of colon cancer to currently available agents, 5-FU alone and 5-FU-based combination chemotherapy are not expected to achieve great success [3, 4]. Therefore, new therapeutic strategies are urgently needed. Currently, combination chemotherapy and adjuvant chemotherapy have achieved great success in the treatment of malignant disease. A combination of conventional chemotherapies with new therapies directly targeted against the molecular changes in colon cancer seems to be the most promising strategy.

Focal adhesion kinase (FAK) has appeared to be firmly identified to and take a crucial part of cancers growth. FAK expression was elevated in an assortment of tumors metastatic [5, 6]. In addition, the balance of FAK expression may affect the ability of growth of tumor cells, proliferation, and a balance of the particular FAK inhibitors with cytotoxic may be a promising anticancer treatment [7, 8].

*Lumbricus rubellus, *an earthworm species of the class* Oligochaeta, *belongs to the family* Lumbricidae*. It is widely found throughout the mainland Europe and also worldwide, including Indonesia. Bioactive compounds have been extracted from earthworms in various countries and used as therapeutic materials for thousands of years [9]. Some studies have shown that they have therapeutic properties, such as anti-inflammatory, antioxidant, antitumor, and antibacterial [9, 10]. The therapeutic properties are attributable to their coelomic fluid (CFL), which contains biological substances such as lectin, polysaccharides, proteases, antibacterial peptides, and fibrinolytic enzymes [10].

Their CFL was found to have a cytotoxic effect in HeLa cells after exposure for 48 hours [11]. Antitumor effects also were showed by CFL and inhibited the proliferation of HeLa cells [9–11]. The CFL of* Eudrilus eugeniae* also had a cytotoxic effect on HeLa cells, leukemia cells, and brain tumor cells [12]. In colorectal cancer progression, FAK plays a critical role. It is an oncogenic protein located in the cytoplasm and regulates cell attachment, proliferation, survival, and motility [13]. The activation of FAK also leads to increased expression of cyclin D1 [14]. Integrin-dependent and independent FAK are localized to the nucleus and interact directly with p53 to promote cell proliferation and survival through p53 degradation [15]. A protein downstream of p53 protein such as p21 plays a role in regulating proliferation, apoptosis, and metastasis of cancer cells. Activation of p21 is expected to suppress the progression of colon cancer [16]. Recently, increased Bcl-2 expression was reported to increase intracellular Ca^2+^ (iCa^2+^) in the cytoplasm [17]. Alterations of iCa^2+^ in cancer cells play essential roles in tumor initiation, angiogenesis, progression, and metastasis [18]. The increase in iCa^2+^ appeared to be mediated by a calcium release mechanism. Furthermore, targeting [iCa^2+^] signaling may be a novel strategy to develop more effective treatments for colon cancer. It is signaling of [iCa^2+^] is critical for regulating cell migration and invasion. However, whether it has antitumor activity against colon cancer is not yet clear.

This study showed evidence that the CFL of* L. rubellus* has antitumor properties in vitro. On the basis of the antitumor effect of CFL, we examined the therapeutic potential of the CFL in the inhibition of colorectal cancer. Further analysis revealed that p21 activated by this fluid was associated with reduced FAK and [iCa^2+^].

## 2. Materials and Methods

### 2.1. Collection of Earthworms

The earthworms were collected from a commercial vermin-culture, CV. RAJ Organic-Malang-Indonesia, and maintained in plastic tubs containing decomposed organic matter at Department of Parasitology, Faculty of Medicine, Brawijaya University. The experiments were performed in accordance with the guidelines and approval (No.238/EC/KEPK/10/2017) of the Institutional Animal Care and Use Committee of Brawijaya University and followed institutional requirements concerning the care and handling of animals according to Guiding Principles for the Care and Use of Animals for Scientific Purposes in the Institutional Animal Care and Use Committee (IACUC).

### 2.2. Coelomic Fluid Collection by Heat and Cold Shock Method

The earthworms were washed in distilled water and then placed in a glass funnel. Worms (20 grams) were kept immersed under 25 ml of warm water (45-50°C) in a glass beaker. This heat shocks method yielded 0.5 ±0.25 ml of coelomic fluid. Heat shock was used as well as in a similar way ice cubes in the cold shock method. All these fluids were subsequently employed for further experimentation. The treatment was alternated at a gap of five minutes to overcome the shock effect. The fluid released was collected into the tubes.

### 2.3. Coelomic Fluid Precipitation

Coelomic fluid was precipitated by Ammonium sulfate and was slowly added to the coelomic fluid to make a final concentration. The pellet collected by centrifugation was resuspended in 20 mM Tris-HCl (pH 8.0) followed by centrifugation. Then, the pellet was resuspended in cold acetone by adding the acetone slowly, followed by 20-minute incubation. The remaining acetone was removed from the pellet by centrifugation. Protein concentrations were measured using the Bradford method with bovine serum albumin (BSA) used as the standard.

### 2.4. Maintenance and Culture of HT-29 Cells

The cell line was purchased from ATTC, USA. Human colon cancer cell line HT-29 was maintained in McCoy medium supplemented with 10% FBS (Fetal Bovine Serum) FCS, 2 mM glutamine, 100 IU/mL penicillin, and 0,1 mg/mL streptomycin. The cells were grown in the humidified CO2 incubator (Binder's, USA) at 5% CO2 level at 37°C. Passage of cells was carried out by 0.05% trypsin-EDTA at 70-80% confluence. Cell line HT-29 was cultured at concentration 5x10^5^ cells/ ml at 24-well plate, until confluent state reached 80%. The cells were cryopreserved with DMSO and stored at -80°C for further stocks.

### 2.5. Experimental Groups and Treatments

HT-29 cells were treated with CFL and 5-fluorouracil for 24 hours in different doses. HT-29 cells were cultured and divided into six groups: group 1 was control, groups 2-5 were treated with 5-fluorouracil, groups 3-5 were treated with either CFL 5, 10, or 20 *µ*g/ml immediately after 5-fluorouracil, and group 6 was treated with CFL 20 *µ*g/ml.

### 2.6. Apoptosis Analysis

Apoptosis cells were determined quantitatively by flow cytometry using the annexin V-conjugated FITC (eBioscience) following the manufacturer's protocol. Briefly, HT-29 cells were treated with 5-fluorouracil alone or with CFL for 24 hours, and cells were harvested, washed with PBS, and incubated with annexin V (FITC) and Propidium Iodide for cell staining in binding buffer (room temperature, 15 minutes in the dark). The stained cells were analyzed using Cell Quest software.

### 2.7. Flow Cytometry Analysis for FAK and p21

FAK and p21 percentages were analyzed by flow cytometry according to Zuryn et al. (2014) [19]. Briefly, cells grown in 6-well plates were harvested, washed with PBS, centrifuged (5 minutes, 300 × g), and fixed with 1% methanol-free, ultrapure formaldehyde. After incubation on ice for 15 minutes, cells were centrifuge for 15 minutes, 300 x g). The cells pellets were permeabilized by the addition of 1 ml of ice-cold 50% (v/v) methanol. After that, cells were incubated for 30 minutes on ice, washed twice with cold PBS, and resuspended in 0.5% bovine serum albumin (BSA; Sigma-Aldrich) for 10 minutes. For intracellular staining, the cell suspensions were transferred into flow cytometric tubes containing 20 *μ*l of FITC conjugated monoclonal anti-human p21 or FAK (Santa Cruz Biotechnology, Inc.), and 200 *μ*l of 0.5% BSA. Following a 45-minute incubation (4°C, in the dark) and washing with PBS, the cells were centrifuged (5 minutes, 500 × g) to wash off excess antibody and resuspended in 200 *μ*l of PBS for flow cytometric analysis on FACS Calibur (Becton-Dickinson). Cell Quest software (Becton-Dickinson) was used to calculate the percentage of p21 or FAK positive cells.

### 2.8. Measurement of Intracellular Calcium Using Fluorescence Microscopy

Measurement of intracellular calcium accumulation Fluo 3-AM (Bioscience, USA), a fluorescent probe, was applied to determine intracellular calcium. Briefly, cells were washed twice with PBS at 800 g for 10 minutes each and resuspended in McCoy medium at 1×10^5^ cells/ml. The cell suspension was plated on polylysine coated slides and allowed to adhere for 10 minutes. HT-29 cell were loaded with BSA 1% and incubated for 60 minutes at room temperature. After loading BSA 1%, 10 *µ*M fluo-3/AM was added and incubated for 60 minutes at 37°C. After the incubation, samples were fixed for 60 minutes with 4% PFA and washed twice with PBS for 5 minutes each time. Fluo-3/AM were observed with 515nm wave and the Ca^2+^ were analyzed with imageJ software.

### 2.9. Statistical Analyses

Data are expressed as mean ± SEM and statistically evaluated with one-way ANOVA followed by the Bonferroni* post hoc* test. Results were expressed as means ± standard deviation. Differences between means were determined by the Mann-Whitney U test (with the level of significance established at p<0.05). Statistical analysis was performed with SPSS software version 20 (IBM Corporation, USA).

## 3. Results

### 3.1. CFL in Combination with 5-FU Induced Apoptosis of HT-29 Cells

**Figure 1:**
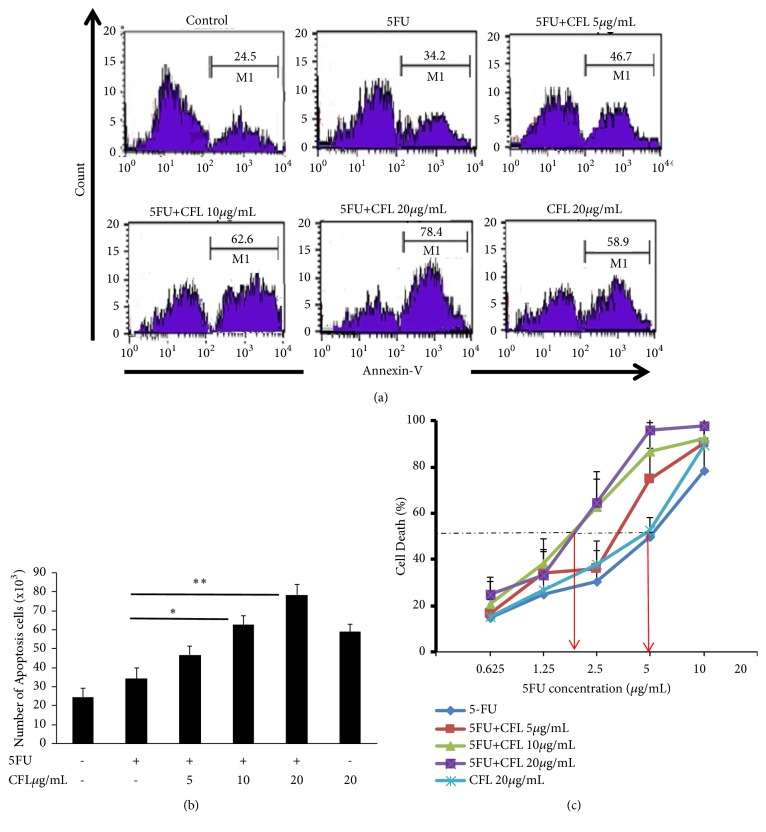
The combination therapy of CFL with 5-fluorouracil induced apoptosis in HT-29 cell line. (a) HT-29 cell line was cultured and divided into six groups: group 1 was without treatment (control); group 2 was treated with 5-fluorouracil, groups 3-5 were treated with either CFL 5, 10, or 20 *µ*g/ml immediately after 5- fluorouracil, and group 6 was treated with CFL 20 *µ*g/ml. The number of apoptosis cells showed that combination treatment has higher number than single treatment group, respectively (p<0.05; p<0.05; p<0.05). Red lines (isotype control). (b) Number of annexin-V positive cells indicated apoptosis cells. (c). Dose response of CFL showed cytotoxic synergistic effects (results shown are mean ± SD, with n =4 *∗*p < 0.05, *∗∗*p < 0.001.

The combination of CFL and 5-FU exerts an enhanced apoptosis effect on HT-29 cells. As for confirming the improved apoptosis effect of the cotreatment, we assessed the number of apoptosis of HT-29 cells (Figure 1). This study had definite effect in apoptosis after treatment with 5-FU alone or in combinations with f 5, 10, and 20 *μ*g/mL CFL. After 24 hours of treatments, the apoptosis rate significantly increased by half or more (p<0.001). Treatment with 5 *μ*g/mL 5-FU only slightly increased the number of apoptosis cells (p<0.05)

### 3.2. Reduced FAK Expression Induced by CFL Combined with 5-FU

**Figure 2:**
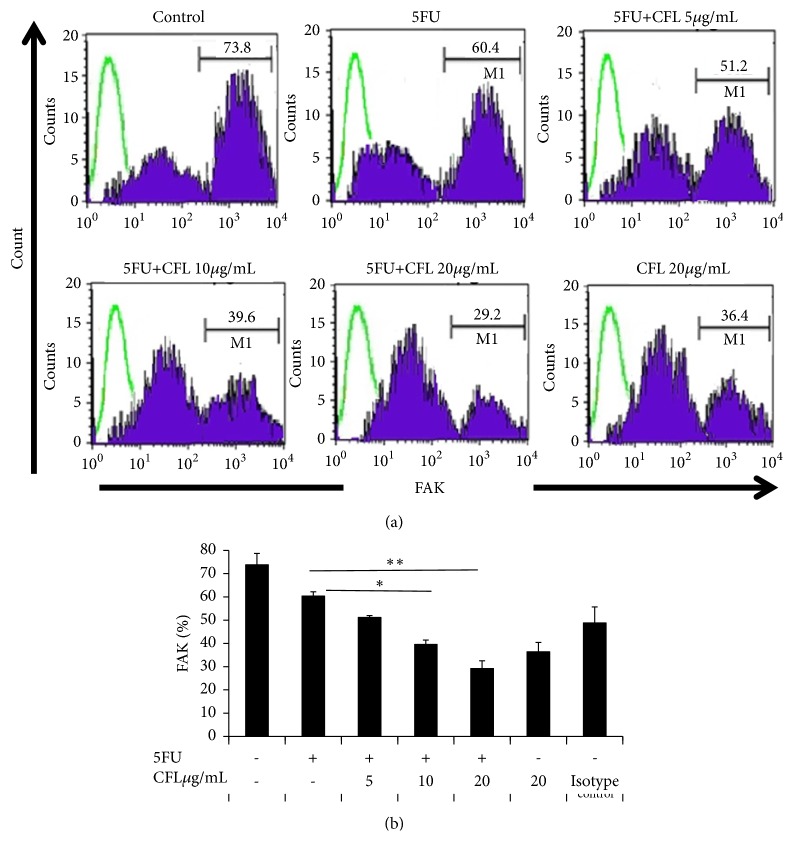
The combination therapy of CFL with 5-fluorouracil reduced the percentage of FAK protein level in HT-29 cells. HT-29 cells were cultured and divided into six groups: group 1 was without treatment (control); group 2 was treated with 5-fluorouracil (5FU), groups 3-5 were treated with either CFL 5, 10, or 20 *µ*g/ml added immediately after 5-fluorouracil, and group 6 was treated with CFL 20 *µ*g/ml. The percentage of FAK of 5FU group was significantly different from the three groups of 10 *µ*g/mL (p<0.05) and 20 *µ*g/mL (p<0.05) and CFL groups (p<0.05). (a) Results are representative of four independent experiments. Green lines show isotype control IgG2b. (b) Number of FAK positive cells. Results shown are mean ± SD, with n =4 replicates in each group. *∗*p<0.05, *∗∗*p < 0.001.

As to confirm the enhanced anticancer effect of the cotreatment, we assessed the expression level of FAK. FAK expression was determined by using flow cytometry. As shown in Figure 2, CFL alone decreased the percentage of FAK expression. The combination of 5-FU and CFL exert an enhanced anticancer effect on HT-29 cells. The representative images shown in Figure 2 demonstrated a clear decreased FAK expression after treatment with CFL alone or in combinations with CFL and 5-FU. After 24 hours of treatments, the FAK expressions significantly decreased by half (p<0.05). Treatment with 5-FU only slightly reduced the expression of FAK (p<0.05). When the combination of CFL and 5-FU was applied, the percentage of FAK expression was lower than 5-FU or CFL alone (p<0.05), which indicated that FAK expression was decreased via CFL and 5-FU combinations (Figure 2).

### 3.3. Reduced iCa^2+^ Expression Induced by CFL Combined with 5-FU

**Figure 3:**
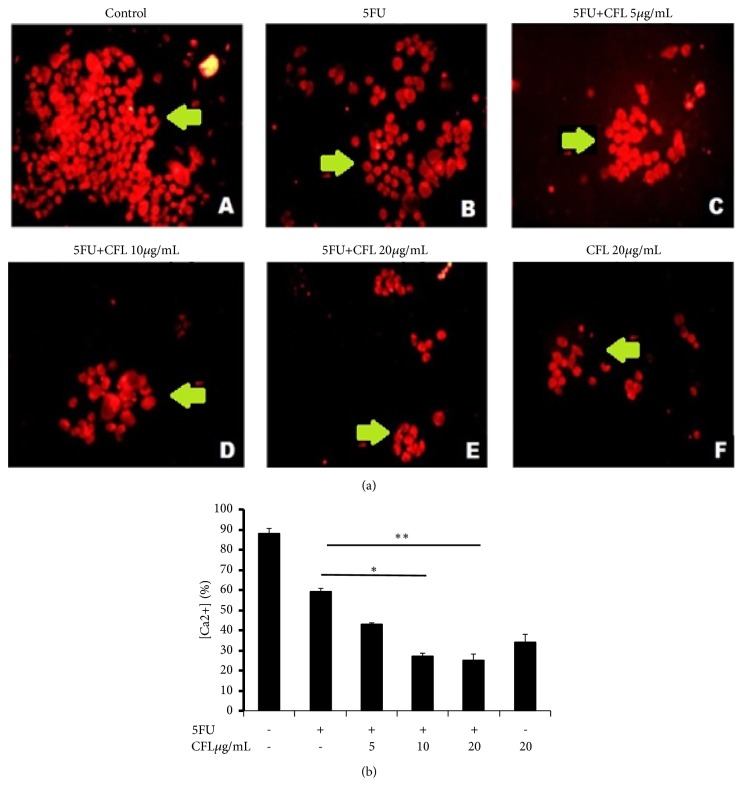
The combination therapy of CFL with 5-fluorouracil reduced the expressions of Ca^2+^ intracellular level in HT-29 cells. (A) Control group. (B) Group was treated with 5FU only. (C), (D), and (E) HT-29 cells were treated with CFL 5, 10, and 20 *μ*g/mL, respectively, after 5-fluorouracil, (F) HT-29 cells were treated with CFL only. Green arrow: Ca^2+^ intracellular positive cell. All images were magnified at representative 4 independent experiments (G). Number of Ca^2+^ intracellular positive cells. The result of [iCa^2+^] expression also showed that combination groups have lower expression than 5FU only group, respectively (p<0.05; p<0.05; p<0.05). ^*∗*^p-value < 0.05; ^*∗∗*^p-value < 0.001.

Since the administration of CFL and 5-FU combinations caused change in expression of FAK protein, we hypothesized that CFL and 5-FU combinations could have effects on the decrease of iCa^2+^ concentration. We analyzed the effect of CFL and 5-FU combinations in iCa^2+^ concentration using immunofluorescence. As shown in Figure 3, the number of iCa^2+^ expression in control group was significantly increased (p<0.05). The treatment of CFL with or without 5-FU combinations can decrease significantly iCa^2+^ concentration compared with 5-FU alone (p<0.05). In this study, based on Figure 3 combination of 10 *μ*g/ml CFL and 5 *μ*g/ml 5-FU was able to decrease the percentage of FAK compared with 5-FU only.

### 3.4. Combination of 5-FU and CFL Increased p21 Presentation in HT-29-Cell Line

**Figure 4:**
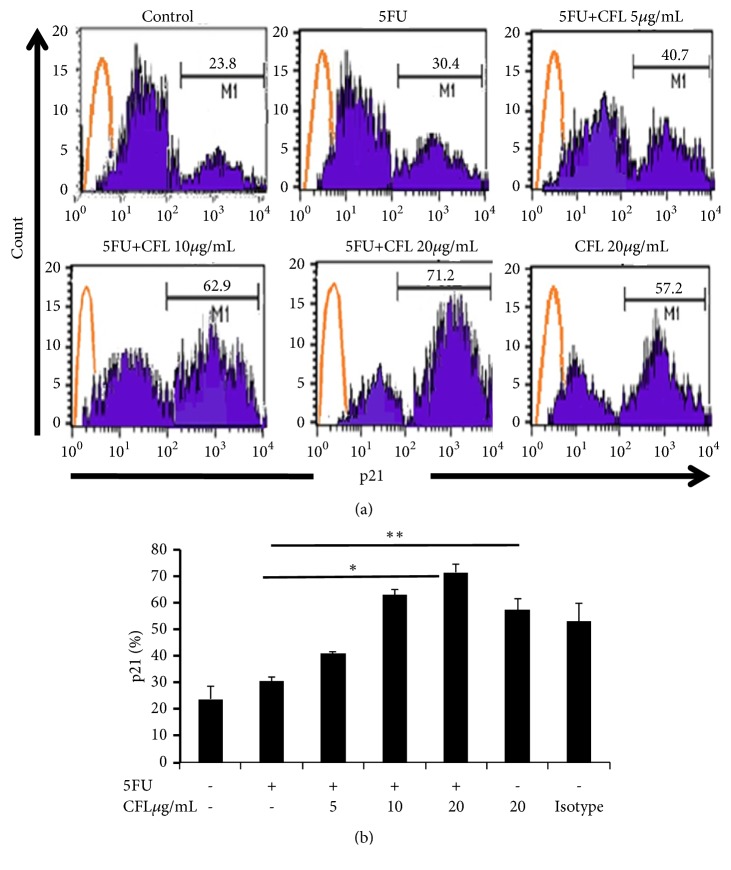
CFL combined with 5-FU reduced p21 expressions of HT-29. The expression of p21 was assessed to evaluate the antimetastatic effect of CFL combined with 5-FU. (a) The percentage of p21 positive cells was analyzed using a FACS Calibur flow cytometer (BD Biosciences). Numbers represent the percentages of p21 positive cells were shown, orange lines (isotype control IgG2b). Results are representative of four independent experiments. (b) The percentage of p21 positive cells was shown inside the panels. Results shown are mean+SD, with n=4 replicates in each group. ^*∗*^p-value < 0.05; ^*∗∗*^p-value p< 0.001.

The effect of CFL on HT-29 cells treated with combination therapy: p21 expression was determined by using flow cytometry. The percentage of p21 for each group was significantly increased (p< 0.05). The percentage of p21 between control and treatment groups was significantly increased (p<0.05) and CFL (p<0.05) (Figure 4). In this study, CFL level significantly influenced the increasing of p21.

## 4. Discussion

In this study, we investigated the enhanced antimetastatic effect of 5-FU on HT-29 cells when combined with CFL. The results showed that calcium-mediated FAK protein is involved in the reduction of p21 signaling. Treatment of HT-29 cells with the combination of 5-FU and CFL reduced FAK expression and [iCa2+] levels and showed antitumor ability through activation of p21 protein. Previously, some proteins and peptides with antitumor activities have been found in CFL [20–22].

[iCa2+] regulates FAK protein signaling, which is involved in cancer cell survival signaling. [iCa2+] levels in cancer cells are affected by changes in Ca2+ transporter, channel, and pump [21]. Changes in the expression or activity of Ca2+ channels and pumps could promote role in cancer [23].

In cancer, the antiapoptotic protein Bcl-2 is commonly deregulated and appears to modulate IP3K. The best characterised Ca2+ store is the ER which houses IP3- and ryanodine-sensitive Ca2+ channels [21–23]. Moreover, Bcl-2 can decrease the sensitivity of the mitochondrial Ca2+ uptake process as well as reducing the ability of Ca2+ to induce apoptosis [24–26]. The influx of Ca2+ through plasma membrane channels, such as calcium release-activated calcium channel protein 1 (ORAI1) and transient receptor potential subfamily V member 6 (TRPV6) cation channel, results in increased Ca2+ entry and induces cell proliferation [24]. Our results suggest that the two pathways activated by CFL induced p21 activation; subsequently, a PI3K pathway leads to the sensitization of (transient receptor potential subfamily V member 1 (TRPV1) cation channel. This sensitization increased [iCa2+] via voltage-gated channels [24, 25]. In the same way, the increase in [iCa2+] will activate calpain, which degrades FAK and enhances the motility of cancer cells [25]. Calpain is a cysteine protease which plays a critical role in focal adhesion dynamics in motile cells [26].

In malignant cells, iCa2^+^ regulates the molecular machinery of cell migration. FAK plays a key role in apoptosis and migration of cancer cells [27]. FAK inhibitors have been shown to reduce tumor metastasis. Due to the role of FAK in EMT, calpain-dependent proteolysis of FAK may have the potential to suppress cancer cell motility [26, 27]. Activated FAK plays an important role as a key signal mediator in tumor progression and metastasis. The increasing of FAK expression contributes to metastasis and tumor proliferation [27, 28].

These results suggest that by increasing [iCa2+] and activation of IP3, the efficacy of the combination of CFL with 5-FU could be increased resulting in a higher rate of apoptosis. Tumorigenesis is associated with the inactivation of p21, a protein downstream from p53 that regulates metastasis.

Several promising colon cancer studies revealed that FAK is associated with p21. FAK has been shown to localize in the nucleus and interact directly with p53 to promote cell proliferation and survival through p53 degradation by mdm2 [28]. This result suggested that the combination of CFL and 5-FU could increase p21 expression through the inhibition of FAK protein. The inhibition of FAK protein led to increase in p21 expression through p53 pathway [29].

The phosphorylation of FAK such as [iCa2+] at its serine residues, as shown for HT-29 cells with [iCa2+], is activated and leads to the activation of FAK by phosphorylating FAK. This study showed that a different stimulus was able to increase p21 levels irrespective of FAK status. Thus, FAK functions in these advanced cancer cells were to suppress p53-dependent transcription of p21 by degradation. In addition, we found that FAK interacts with several binding partners, such as p53 and p21 [10, 29], FAKs link to apoptotic pathways and that p21 inhibits FAK transcription in vitro. There was an interaction between p53 and FAK as an example of focal adhesion protein functioning in the signaling between extracellular matrix and nucleus. FAK can also block p53 transcriptional activity of p21 [10, 30, 31]. Thus, this study suggested that p21 can induced apoptosis through downregulation of metastasis-related genes, such as FAK. We have shown recently that p21 can downregulate FAK expression inhuman cancer cells [28, 31]. HT-29 cells express a p53 mutated form, which can still induce p21 and cell cycle arrest. It was therefore somewhat surprising when several tumor-derived mutants of p53 were demonstrated to interact with both p63 and p73 [32, 33]. This interaction has been invoked to explain many of the gain-of-function effects of mutant p53. The mutant of p53 can bind to p63 and inhibit p63-transcriptional activity [34]. This report explains that direct interaction of mutant p53 with p63 was able to transactivate p21. Then, we demonstrated a high correlation between FAK overexpression and p21 in breast cancer cells.

Our results showed that a decrease in the percentage of FAK was related to the dose of CFL. This result proves that CFL administration was able to reduce FAK protein expression. This further suggests that 5-FU as a single therapeutic agent has not been sufficient for colon cancer treatment. The increase in p21 levels was related to the CFL dose, proving that CFL treatment can affect p21 protein levels.

Ca2^+^ increase in cancer cells is affected by changes in the Ca2^+^ transporter, channel, and pump [25, 32]. Ca2^+^ concentrations can be used as a therapeutic target and inhibiting Ca2^+^ transporter, channel, and pump would regulate Ca2^+^ levels [35, 36]. The results indicated a strong correlation between FAK and Ca2^+^. According to Sundaramoorthy et al. [37], it was revealed that increased intracellular calcium in cancer cells activates the cysteine protease of calpain and induces the cleavage and transfer of FAK into the nucleus [35, 37]. In colorectal cancer, Bcl-2 prevents apoptosis by suppressing p53 [32–34]. p53 activates* Kruppel like factor-4* (KLF4) transcription thereby causing a decrease in the level of transcriptional factor C*yclin-dependent kinase inhibitor 1A* (CDKN1A), which is a p21 protein-coding gene; eventually, p21 levels decrease [37–40]. CCF-1 acting as an analog of TNF is expected to suppress Bcl-2 thus increasing the expression of p21 and suppressing FAK and iCa2^+^. Based on this finding, Bcl-2 plays a significant role in decreasing p21 and activating FAK and iCa2^+^. Furthermore, by inhibiting Bcl-2 in the coelomic fluid using TNF analogs [39–42], activation of FAK and iCa2^+^ can be suppressed.

## 5. Conclusions

Our results suggest that FAK can be a new therapeutic target for colon cancer, and the development of selective FAK inhibitors may be a promising way to enhance CFL chemosensitivity in colon cancer.

## Data Availability

These data used to support the findings of this study are available from the corresponding author upon request.

## Abbreviations

5-FU: * 5-Fuoro Uracil*
Bcl-2: * B-cell lymphoma* 2
CCF-1: Coelomic Cytolytic Factor-1
CDKN1A: * Cyclin-dependent kinase inhibitor 1A*
CFL: * Coelomic fluid Lumbricus*
dTMP: * Deoxythymidine monophosphate*
FAK: * Focal adhesion kinase*
IP_3_: * Inositol 1,4,5-trisphosphate*
KLF4: * KLF4 Kruppel like factor-4*
ORAI1: * Calcium release-activated calcium channel protein 1*
TNF: * Tumor Necrosis Factor*
TRPV1: * Transient receptor potential cation channel subfamily V member* 1
TRPV6: * Transient receptor potential cation channel subfamily V member* 6
TS: * Thymidylate synthetase*.
